# B cell deficiency promotes the initiation and progression of lung cancer

**DOI:** 10.3389/fonc.2022.1006477

**Published:** 2022-09-29

**Authors:** Han Wu, Chen Chen, Lixing Gu, Jiapeng Li, Yunqiang Yue, Mengqing Lyu, Yeting Cui, Xiaoyu Zhang, Yu Liu, Haichuan Zhu, Xinghua Liao, Tongcun Zhang, Fan Sun, Weidong Hu

**Affiliations:** ^1^ College of Life Sciences and Health, Wuhan University of Science and Technology, Wuhan, China; ^2^ Institute of Biology and Medicine, Wuhan University of Science and Technology, Wuhan, China; ^3^ Department of Biological Repositories, Zhongnan Hospital of Wuhan University, Wuhan, China; ^4^ College of Science, Wuhan University of Science and Technology, Wuhan, China; ^5^ Department of Thoracic Surgery, Zhongnan Hospital of Wuhan University, Wuhan, China

**Keywords:** B cell, CAR-T, lung cancer, initiation, progression

## Abstract

Currently commercialized CAR-T cell therapies targeting CD19 and BCMA show great efficacy to cure B cell malignancies. However, intravenous infusion of these CAR-T cells severely destroys both transformed and normal B cells in most tissues and organs, in particular lung, leading to a critical question that what the impact of normal B cell depletion on pulmonary diseases and lung cancer is. Herein, we find that B cell frequency is remarkably reduced in both smoking carcinogen-treated lung tissues and lung tumors, which is associated with advanced cancer progression and worse patient survival. B cell depletion by anti-CD20 antibody significantly accelerates the initiation and progression of lung tumors, which is mediated by repressed tumor infiltration of T cells and macrophage elimination of tumor cells. These findings unveil the overall antitumor activity of B cells in lung cancer, providing novel insights into both mechanisms underlying lung cancer pathogenesis and clinical prevention post CAR-T cell therapy.

## Introduction

CAR-T cell therapy represents the most revolutionary breakthrough in treatment of hematopoietic cancers in the new century (1). At present, there are eight products of CAR-T cells marketed all over the world with six targeting CD19 and two BCMA to cure B-cell leukemia/lymphoma and multiple myeloma, respectively (2, 3). In addition to strongly expressed in B cell malignancies, both molecules are constitutively and exclusively expressed on naïve and memory B cells. Consequently, current CAR-T cell therapy results in severe loss of both malignant and normal B cells in most tissues and organs, in particular lung, the major accumulation site of CAR-T cells by intravenous infusion (1–3). However, the long-term effect of normal B cell depletion from lung frequently exposed to environmental risks is unknown.

B cell is one of the most abundant immune cells in lung tissues under both physiological and pathological conditions, including lung cancer (4, 5). The main function of B cell comprises antibody production for humoral immunity, antigen presentation for T cell immunity, and immune regulation. However, different from T lymphocytes, the role of B lymphocytes in cancers is in debate (6–8). In murine pre-malignancy models, failure in immune cell recruitment and tumorigenesis in K14-HPV16 mice (T and B cell-deficient) can be overcame by adoptive transfer of B cells or serum from HPV16 mice to induce strong infiltration of innate immune cells and malignant progression, emphasizing the indispensable role for B cell in establishment of chronic inflammatory state to promote carcinogenesis (9). In line with these findings, B cell depletion by administration of anti-CD20 antibody is capable of preventing premalignant dysplasia in K14-HPV16 mice with resultant reduced levels of serum IgG and immune cells in neoplastic site (10). Similarly, compared to B cells with selective TNFα deletion, adoptive transfer of B cells from wild type mice into TNFα knockouts notably stimulates papilloma development in DMBA/TPA murine model of skin tumorigenesis, highlighting the tumor-promoting function of Breg, the known source of TNFα (11). Meanwhile, IFNγ secretion and T cell infiltration are elevated in TNFα knockout mice (11). Consistently, B cells not only induce a non-protective humoral immune response, but also inhibit CD4^+^ T cells to mount CTL-mediated tumor immunity (12).

The pro-tumor role of B cells is also observed in human cancers. An increase of tumor-infiltrating B cells is associated with poor prognosis and survival in patients with metastatic ovarian cancer (13, 14). In tumor tissues, STAT3 activation in B cells is positively associated with tumor angiogenesis (15). Moreover, partial B-cell deletion by rituximab leads to repressed tumor burden in half patients with advanced colorectal cancer (16). These studies support the positive role for B cells in fostering malignant transition and progression in both murine and human cancers.

On the other hand, the antitumor role of B cells is found in murine established tumor models. Anti-CD20-mediated B cell depletion exacerbates primary tumor burden and pulmonary metastasis in B16 melanoma model (17), whereas adoptive transfer of CpG-primed B cells inhibits tumor progression of melanoma in B-cell deficient mice (18). Accordingly, increased tumor growth and metastasis is observed in mice bearing 4T1 breast tumor by administration of anti-CD20 antibody, with enriched Breg abundance and impaired T cell activity, which can be reverted by adoptive transfer of CpG-activated B cells (19). The tumor-inhibiting role of B cells involves both direct killing of tumor cells through FasL/Fas and granzyme B/perforin pathways and indirect eradication of tumor by promotion of tumor infiltration of T cells, complement-dependent tumor cell lysis, and antibody-mediated ADCC and phagocytosis of tumor cells (20).

The antitumor role of B cells is also reported in human cancers in literature. B cell frequency in tumor is positively correlated with patient survival, representing a novel prognostic cellular biomarker (21). Moreover, the presence of B cells in tertiary lymphoid structure (TLS) is positively associated with protective tumor immunity, including high levels of naïve, memory, and activated T cells, and low levels of exhausted T cells and Tregs (22–24).

In summary, the role of B cells in tumor initiation and progression is controversial in both human and murine cancers, in particular lung cancer. Given malignant and normal B cell depletion by current CAR-T cell therapy, to explore its consequence is urgently needed to improve clinical treatment and/or prevention post therapy. In the present study, the prognostic role of B cells in lung cancer was extensively analyzed with public databases. Then, the dynamic changes of B cell frequency in lung tissues and lung tumors were examined during lung tumorigenesis. The effect of B cell deficiency on tumor infiltration of immune cells, T cell activation, tumor initiation and progression was investigated in endogenous lung tumor model. These findings about B cell function will provide novel insights into both lung cancer pathogenesis and clinical prevention post CAR-T cell therapy.

## Materials and methods

### Public data mining

The gene expression data of B cell specific markers (CD19, CD79A, CD79B, BLK, and CD20/MS4A1) and clinical characteristics in human lung cancer were retrieved from The Cancer Genome Atlas (TCGA) database in Xena website (http://xena.ucsc.edu). The mean value of these five B cell specific markers was utilized as B cell set for correlation analysis. Individual B cell specific marker was also analyzed. The association of immune infiltrates with clinical outcomes was determined by Kaplan-Meier Plotter (http://kmplot.com/analysis/index.php?p=service&cancer=lung) and TIMER (http://timer.cistrome.org/) as described (25). All information in detail was indicated in Figure Legends.

### Mouse and tumor cell

FVB/N and BALB/c mice originally from Beijing HFK Bioscience Co., Ltd were housed in pathogen-free conditions and used according to protocols approved by the Animal Ethics Committee of Wuhan University of Science and Technology. To induce endogenous lung tumor, 1 g/kg body weight of urethane was intraperitoneally injected once a week for six consecutive weeks in 6-week-old female wildtype mice. After 6-week tumor initiation and 6-week tumor progression, the urethane-treated mice were sacrificed and/or kept for lung tumor analysis and immune profiling at the indicated time points (26). For B cell depletion, anti-CD20 antibody (200 µg per mouse) was administrated through intravenous tail vein injection every three weeks, starting two days before urethane treatment. An isotype control antibody (Ctrl) was included for comparison. For spontaneous lung tumor, female FVB/N wildtype mice were maintained for 24 months and sacrificed for tumor examination and immune cell analysis. For syngeneic xenograft, murine lung tumor cells (LAP0297 and MAD109, 10^6^ cells per mouse) were subcutaneously inoculated (sc) in the right flank or intravenously injected (iv) through tail vein. Then, tumor tissues or lung tissues were collected for immune cell analysis at the indicated time as described (27).

### FACS analysis

The single cell suspensions from fresh lung tissues and lung tumors were blocked with αCD16/CD32 and stained with the antibody against cell surface antigen. If needed, the cells were then fixed with paraformaldehyde (2%), permeablized and incubated with antibodies against intracellular antigens. For nuclear staining, Permeabilization/Fixation buffer was utilized. For IFNγ staining, cells were treated with phorbol 12-myristate 13-acetate (PMA, 50 ng/ml), ionomycin (1 μM), brefeldin A (BFA, 3 μg/ml) and monensin (2 μM) for 4 hours before they were collected for staining. Data were acquired by Accuri C6 or LSRFortessa I and analyzed by FlowJo software as described (28, 29). In brief, CD45 was included as a marker to distinguish immune cells from other cells in cell suspensions both *in vitro* and *in vivo*. Immune cells (CD45^+^) were gated with the indicated markers as follows. Lymphocytes were gated for CD4^+^ T cells (CD3^+^CD4^+^CD8^-^), Treg cells (CD3^+^CD4^+^Foxp3^+^CD25^+^), CD8^+^ T cells (CD3^+^CD4^-^CD8^+^), NK cells (NKp46^+^CD3^-^), and B cells (B220^+^CD3^-^). B lymphocytes were further classified into Breg cells (B220^+^CD1d^+^CD5^+^), memory B cells (B220^+^IgD^-^IgM^+^CD38^+^), and plasma cells (B220^-^CD138^+^). Myeloid cells were gated sequentially as follows: neutrophils (CD11b^+^Ly6G^+^), macrophages (Mac: MerTK^+^CD64^+^; AM: CD11c^+^CD11b^-^; IM: CD11c^-^CD11b^+^), dendritic cells (MHC-II^+^CD11c^+^), monocytes (CD11b^+^SSC^lo^). Tumor cells were gated as CD45^-^EpCAM^+^ cells. All antibodies used for FACS analysis were shown in **Supplementary Table S1**.

### Immunohistochemistry assay

Mouse lung tissues and lung tumors were excised, fixed in formalin, embedded in paraffin, and cut into 4-μm-thick sections. Sections were subjected to sequential incubations with the indicated primary antibodies, biotinylated secondary antibodies and streptavidin-horseradish peroxidase (HRP) as described (29). Images of the staining were analyzed using the image J software. The data represented were from five mice per group, with over 500 cells counted in each mouse. Antibodies used for IHC assay were listed in **Supplementary Table S1**.

### Statistical analysis

Two tailed, unpaired Student’s *t* test was employed to assess significance of differences between two groups. Ordinary one-way ANOVA was performed to analyze the significance of differences among multiple groups. Chi-square test was carried out to determine the association between clinical characteristics. Log-rank test was used to compare survival between groups. All data are represented as bars (means ± SEM) with sample dots. The experimental replication and sample numbers for tumor analysis, FACS, and immunohistochemistry were indicated in FIGURE or FIGURE LEGENDS. The *p* values were indicated as *p < 0.05, **p < 0.01, ns, not statistically significant. The *p* values < 0.05 and 0.01 were considered statistically significant and highly statistically significant, respectively.

## Results

### Tumor-infiltrating B cells are positively associated with patient survival in lung cancer

**Figure 1:**
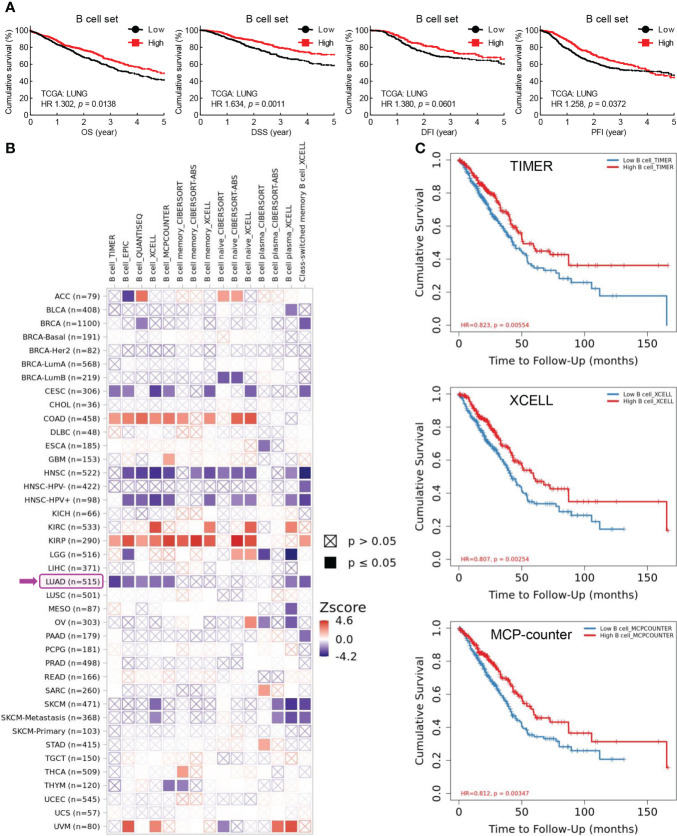
Tumor-infiltrating B cells are positively associated with patient survival in lung cancer. **(A)** TCGA LUNG data showing positive correlation between B cell frequency in lung tumor and overall survival (OS), disease specific survival (DSS), disease free interval (DFI), and progression free interval (PFI). B cell set comprises five specific B cell markers (CD19, CD79A, CD79B, BLK, and CD20). **(B)** TIMER data showing association between B cell infiltrates and clinical outcome in human cancers. Z-score > 0: increased risk, Z-score < 0: decreased risk. **(C)** Kaplan-Meier curves displaying positive association of B cell infiltrates with patient survival in lung adenocarcinoma (LUAD) by TIMER, XCELL, and MCP-counter algorithms. The infiltration level is equally divided into low and high levels. The hazard ratio (HR) and the log-rank *p* value for Kaplan-Meier curve were shown in plots **(A**, **C)**.

B cells are key lymphocytes to mediate humoral immunity against extracellular pathogens, however, the overall function of these antibody-secreting immune cells in lung cancer remains elusive. To determine the role of B cell in lung cancer, public data from TCGA LUNG cohort were retrieved to examine the association between B cell and patient survival. Initially, five specific B cell markers (CD19, CD79A, CD79B, BLK, and CD20/MS4A1) were chosen as a gene set for B cell characterization in RNA sequencing data of lung tumors. Intriguing, both B cell set and individual specific B cell markers were positively correlated with patient survival, including overall survival (OS), disease specific survival (DSS), disease free interval (DFI), and progression free interval (PFI) (**Figure 1A**; **Supplementary Figure S1**). Data from Kaplan-Meier Plotter also exhibited better overall survival (OS), first progression (FP), and post-progression survival (PPS) in patients with high CD20 expression, compared to that in patients with low CD20 expression (**Supplementary Figure S2A**). Taken together, these results indicate the antitumor role of B cell in lung cancer.

To confirm this opinion, TIMER database was employed to investigate the association between tumor-infiltrating B cells and clinical outcomes. As shown in **Figure 1B**, B cell abundance was a favorable biomarker of decreased risk in lung adenocarcinoma (LUAD), which was further evidenced by Kaplan-Meier curves displaying better survival of patients with high tumor infiltration level of B cells estimated by TIMER, XCELL, MCP-counter, EPIC, and QUANTISEQ algorithms (**Figure 1C**; **Supplementary Figure S2B**). Strikingly, the prognostic role of tumor-infiltrating B cells was only observed in LUAD, but not in lung squamous cell carcinoma (LUSC) (**Figure 1B**). This unexpected finding was substantiated by the fact that high level of B cell set was associated with better survival (OS, DSS, DFI, and PFI) in patients with LUAD but not LUSC (**Supplementary Figure S2C**).

In addition, high level of CD20, the specific marker for B cell, and B cell set in lung cancer was correlated with better overall survival in patients received chemotherapy, improved clinical outcomes of primary and follow-up treatments, and delayed tumor progression, respectively (**Supplementary Figure S3**). Taken together, these data suggest that B cells probably exert antitumor function to inhibit tumor progression and promote patient survival in lung cancer.

### B cell is reduced during lung tumorigenesis

**Figure 2:**
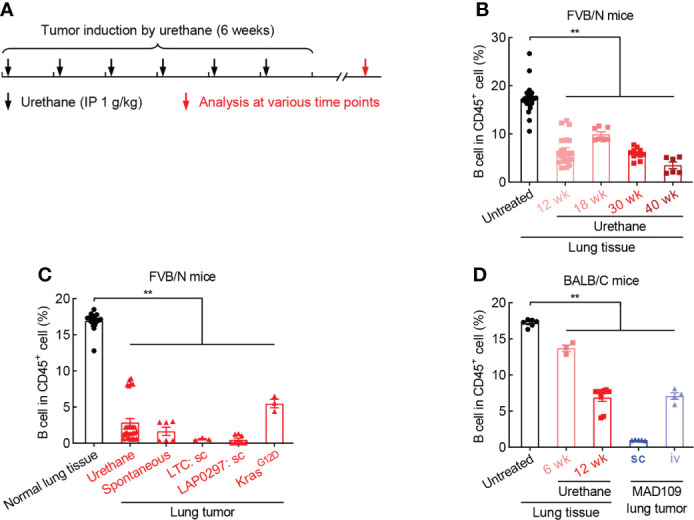
B cell is reduced during lung tumorigenesis. **(A)** Schematic of smoking carcinogen urethane-induced endogenous lung tumorigenesis. **(B)** FACS data showing decrease of B cell in lung tissues exposed to urethane in FVB/N mice. Untreated (n = 24), Urethane 12 wk (n = 23), Urethane 18 wk (n = 7), Urethane 30 wk (n = 11), Urethane 40 wk (n = 6). **(C)** FACS data showing decrease of B cell in multiple lung tumor models in FVB/N mice. Normal lung tissue (n = 17), Urethane-induced lung tumor (n = 28), Spontaneous lung tumor (n = 6), LTC sc (n = 3), LAP0297 sc (n = 8), Kras^G12D^-induced lung tumor (n = 3). **(D)** FACS data showing loss of B cell in both urethane-induced endogenous lung tumor and xenograft models in BACL/C mice. Untreated (n = 6), Urethane 6 wk (n = 3), Urethane 12 wk (n = 9), MAD109 sc (n = 5), MAD109 iv (n = 4). LTC: Lung Tumor Cell which was established from spontaneous FVB/N lung tumor in our laboratory. LAP0297 and MAD109 are lung cancer cell lines originally derived from spontaneous lung tumors developed in FVB/N and BALB/C mice, respectively. sc, subcutaneous injection; iv, intravenous injection (tail vein). Ordinary one-way ANOVA was performed. Data represented means ± SEM **(B**-**D)**. **p < 0.01.

To clarify the role of B cell in lung cancer, smoking carcinogen urethane-induced endogenous murine lung tumor model was employed (**Figure 2A**), which faithfully recapitulates the molecular characteristics and histologic patterns of human lung cancer, and in particular adenocarcinoma associated with tobacco smoking, the most common type of lung cancer that makes up about 40% of all lung cancers. Interestingly, the frequency of B cell was significantly and gradually decreased in lung tissues exposed to urethane (**Figure 2B**). Moreover, urethane-induced lung tumors possessed much fewer tumor-infiltrating B cells in comparison to untreated lung tissues. Consistently, the tumor infiltration of B cells were severely reduced in multiple lung tumor models, including spontaneous lung tumor, subcutaneous lung tumor, and oncogenic Kras^G12D^-induced lung tumor (**Figure 2C**). To further confirm these data found in FVB/N mice, similar experiments were carried out in BALB/C mice, revealing B cell inhibition in both urethane-treated lung tissues and lung tumors (**Figure 2D**). These data suggest that B cell is repressed in both lung tumors and surrounding tissues.

### B cell depletion leads to increased lung tumorigenesis

**Figure 3:**
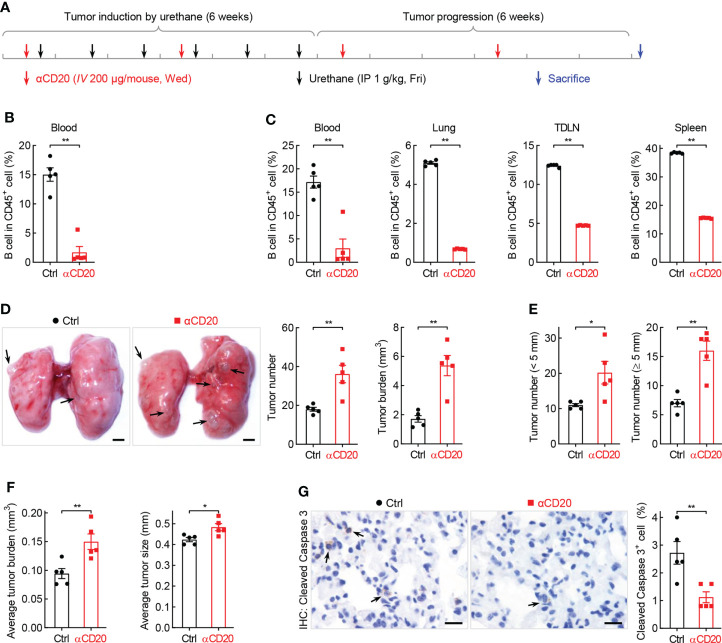
B cell depletion leads to increased lung tumorigenesis. **(A)** Schematic of B cell depletion by αCD20 in smoking carcinogen urethane-induced endogenous lung tumor model. **(B)** FACS data showing B cell depletion by αCD20 in blood before first dose of urethane (n = 5). **(C)** FACS data showing B cell depletion by αCD20 in blood, lung, tumor-draining lymph node (TDLN), and spleen at the endpoint (n = 5). **(D)** Tumor examination showing increased lung tumor number and tumor burden by αCD20-mediated B cell depletion (n = 5). **(E)** Tumor examination showing elevated both small and large lung tumor numbers by αCD20-mediated B cell depletion (n = 5). **(F)** Tumor examination showing enlarged individual lung tumor by αCD20-mediated B cell depletion (n = 5). **(G)** IHC analysis showing decreased tumor cell apoptosis in mice with αCD20-mediated B cell depletion (n = 5). TDLN: mediastinum lymph node. Data shown were representative of two independent experiments with similar results. Scale bar: 1 mm **(D)** and 20 µm **(G)**. Student’s *t* test (two tailed, unpaired) was performed **(B**-**G)**. Data represented means ± SEM **(B**-**G)**. *p < 0.05; **p<0.01.

What is the function of B cell repression in lung tumorigenesis? To address this question, anti-CD20 antibody was utilized to deplete B cells in smoking carcinogen urethane-induced endogenous lung tumor model, which is widely used to study the mechanisms underlying lung tumorigenesis (**Figure 3A**). Firstly, B cells were successfully depleted by anti-CD20 antibody as evidenced by remarkably fewer B lymphocytes in multiple tissues and organs from mice received anti-CD20 antibody, including blood, lung, TDLN (mediastinum lymph node), and spleen, compared to that in tissues from control mice (**Figure 3B, C**). Surprisingly, compared to mice from control group, both lung tumor number and tumor burden were significantly increased in mice underwent B cell depletion (**Figure 3D**). In detail, more small lung tumors and larger average tumor size and burden were identified in mice administrated with anti-CD20 antibody, suggesting enhanced tumor initiation and progression (**Figures 3E, F**). In line with this finding, decreased apoptosis rate was detected in lung tumors from mice treated with anti-CD20 antibody (**Figures 3G**, **Supplementary Figure S4A**). Meanwhile, αCD20-mediated B cell depletion had minimal effect on TDLN weight and body weight (**Supplementary Figures S4B, C**). These data suggest that B cell deficiency promotes the initiation and progression of lung cancer.

### B cell deficiency impairs T cell killing of lung tumor cells

**Figure 4:**
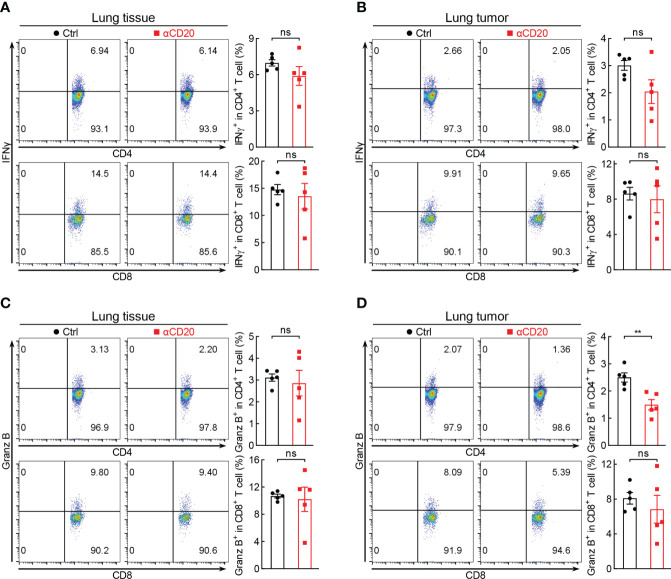
B cell deficiency has negligible effect on T cell activity in lung cancer. **(A, B)** FACS data showing comparable expression levels of IFNγ in both lung tissues and lung tumors between Ctrl and αCD20 groups (n = 5). **(C, D)** FACS data showing comparable expression levels of Granz B (granzyme B) in both lung tissues and lung tumors between Ctrl and αCD20 groups (n = 5). Data shown were representative of two independent experiments with similar results. Student’s *t* test (two tailed, unpaired) was performed. Data represented means ± SEM. **p < 0.01; ns, not statistically significant.

How B cell depletion boosts lung tumorigenesis? Initially, the activity of T cell, the well-known direct killer of tumor cells, was analyzed in the context of anti-CD20 antibody treatment. Unexpectedly, in lung tissues and lung tumors, both CD4^+^ and CD8^+^ T cells expressed comparable levels of IFNγ between B cell-depleted mice and control mice (**Figures 4A, B**). Similar results were obtained with respect to granzyme B (Granz B), another T cell activation marker (**Figures 4C, D**). These data suggest that B cell deficiency has negligible effect on T cell activity in lung cancer.

**Figure 5:**
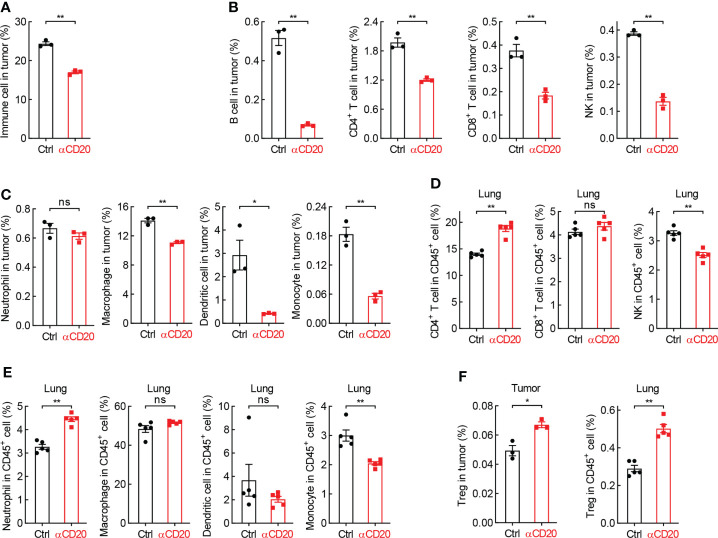
B cell deprivation dampens tumor infiltration of immune cells in lung cancer. **(A)** FACS data showing reduced abundance of immune cells in tumor from mice with αCD20-mediated B cell depletion (n = 3). **(B, C)** FACS data showing decreased tumor-infiltrating lymphocytes **(B)** and myeloid cells **(C)** by αCD20-mediated B cell depletion (n = 3). **(D, E)** FACS data showing differential changes of lymphocytes **(D)** and myeloid cells **(E)** in lung tissue by αCD20-mediated B cell depletion (n = 5). **(F)** FACS data showing augmented Treg cell frequency in both lung tumor (n = 3) and lung tissue (n = 5) by αCD20-mediated B cell depletion. Student’s *t* test (two tailed, unpaired) was performed. Data represented means ± SEM. *p < 0.05; **p < 0.01; ns, not statistically significant.

Although T cell activity unchanged in both lung tissues and lung tumors, the tumor infiltration of immune cells was severely inhibited by B cell depletion (**Figure 5A**). In detail, lymphocytes, including B cell, CD4^+^ T cell, CD8^+^ T cell, and NK, were significantly decreased in tumors from mice treated with anti-CD20 antibody, compared to that in control mice (**Figure 5B**), and so were myeloid cells, including macrophage, dendritic cell, monocyte, but not neutrophil (**Figure 5C**). In parallel, only NK and monocyte were suppressed by αCD20-mediated B cell depletion in lung tissues, whereas CD4^+^ T cell and neutrophil increased (**Figures 5D, E**), indicating differential roles of anti-CD20 antibody in modulating immune cell populations in lung tumors and lung tissues. It was worth noting that Treg cells were remarkably elevated by B cell depletion in all tissues/organs tested, including tumor, lung, TDLN, and spleen (**Figure 5F**; **Supplementary Figure S5**). Taken together, these data suggest that B cell deprivation dampens tumor infiltration of most immune cells, but enhances Treg cells in lung cancer.

### B cell deficiency impedes macrophage elimination of lung tumor cells

**Figure 6:**
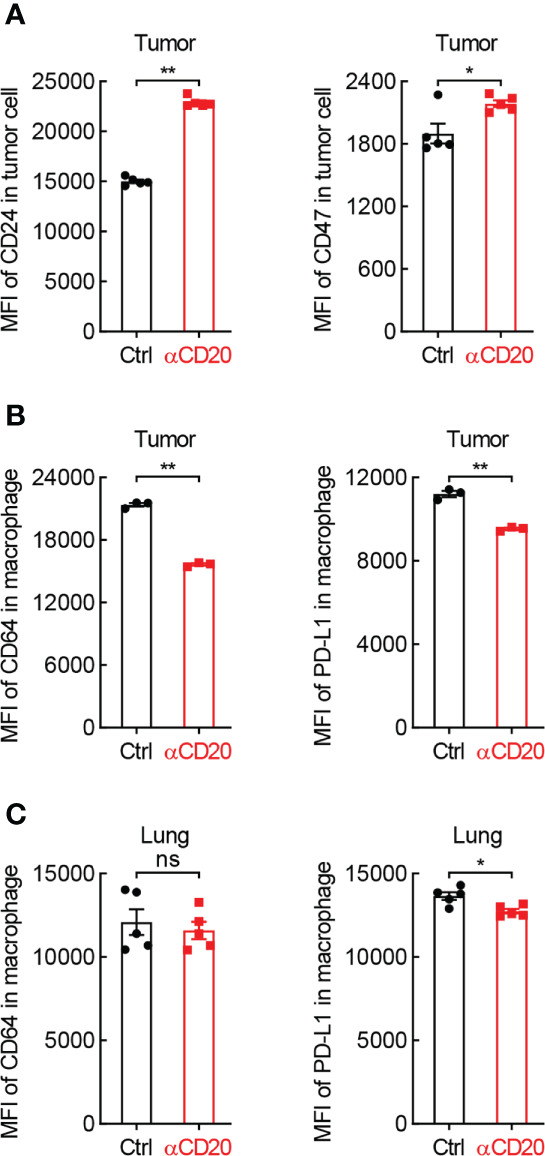
B cell shortage impedes the expression of phagocytosis-related genes in lung cancer. **(A)** FACS data showing elevated expression of CD24 and CD47 on tumor cells from mice with αCD20-mediated B cell depletion (n = 5). **(B)** FACS data showing impaired expression of PD-L1 and CD64 on tumor-infiltrating alveolar macrophages from mice with αCD20-mediated B cell depletion (n = 3). **(C)** FACS data showing reduced expression of PD-L1 but not CD64 on alveolar macrophages in lung tissues from mice with αCD20-mediated B cell depletion (n = 5). Student’s *t* test (two tailed, unpaired) was performed. Data represented means ± SEM. *p < 0.05; **p < 0.01; ns, not statistically significant.

Besides T cell-mediated tumor eradication, macrophage-guided phagocytosis is critical for tumor cell elimination as well. Surprisingly, both CD24 and CD47, two classic ligands of “don’t eat me” signal, were notably upregulated on tumor cells from mice received anti-CD20 antibody (**Figure 6A**), whereas comparable expression levels of their receptors were observed on macrophages, Siglec-10 and SIRPα, respectively (Data not shown). These data indicate that B cell deficiency shifts macrophage’s function towards “don’t eat me” signal in lung cancer.

Given B cells are the essential source of antibodies, signaling molecules responding for antibody-dependent cellular phagocytosis were then examined. Intriguingly, CD64, also known as FcγRI which is a high-affinity receptor for IgG to initiate specific phagocytosis of pathogens and tumor cells, was significantly downregulated on tumor-infiltrating macrophages in anti-CD20 antibody-treated mice, compared to that in control mice (**Figure 6B**). Similar results were achieved in respect of PD-L1 (**Figure 6B**), which was recently identified as a novel stimulator of phagocytosis in alveolar macrophages (28). Moreover, the expression of PD-L1, but not CD64, was abated on macrophages in lung tissues from B cell-depleted mice as well (**Figure 6C**). Taken together, these data suggest that B cell deficiency restrains macrophage-mediated elimination of tumor cells in lung cancer.

### B cell subsets are diminished by CD20 blockade in lung cancer

**Figure 7:**
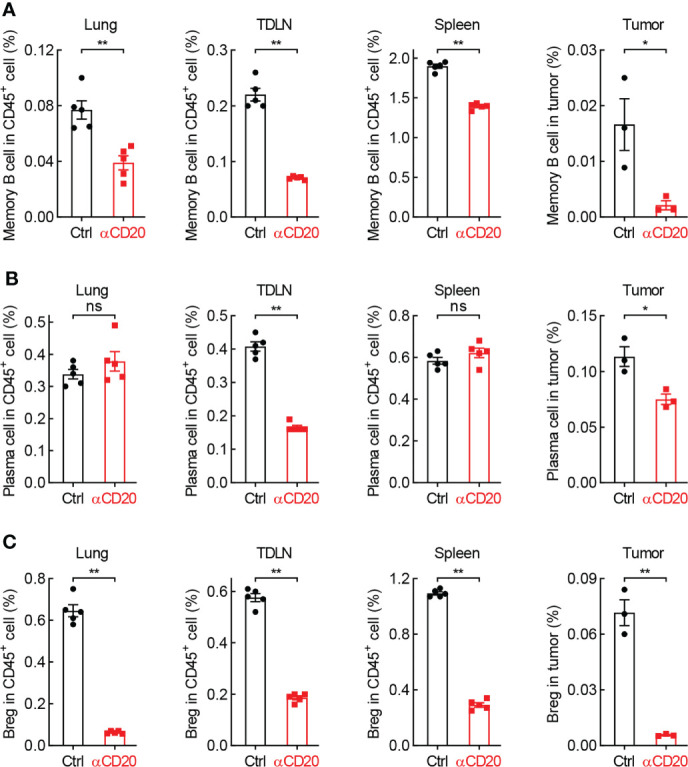
B cell subsets are diminished by CD20 blockade in lung cancer. **(A)** FACS data showing fewer memory B cells in multiple tissues and organs from mice with αCD20-mediated B cell depletion. **(B)** FACS data showing reduced plasma cells in TDLN and tumor, but not lung tissue and spleen from mice with αCD20-mediated B cell depletion. **(C)** FACS data showing decreased Breg cells in multiple tissues and organs from mice with αCD20-mediated B cell depletion. TDLN: mediastinum lymph node. Lung tissue: n = 5; TDLN: n = 5; Spleen: n = 5; Lung tumor: n = 3. Student’s *t* test (two tailed, unpaired) was performed. Data represented means ± SEM. *p < 0.05; **p < 0.01; ns, not statistically significant.

Given many B cell subpopulations in lung tissues and lung tumors (30), the effect of anti-CD20 antibody on some primary B cell subsets was surveyed. As expected, memory B cells and Breg cells, both expressing CD20 molecule, were significantly decreased in various tissues/organs from mice treated with anti-CD20 antibody, including lung, TDLN, spleen, and tumor; however, plasma cells only diminished in TDLN and tumor (**Figure 7**; **Supplementary Figure S6**). Considering the exacerbated lung tumor initiation and progression by B cell depletion, these data suggest that the pro-tumor function of Breg cells is dominated by the antitumor activity of memory B cells and plasma cells in tumor microenvironment, rendering an overall immune suppression by CD20 blockade in lung cancer.

## Discussion

Given the contradictory conclusions of B cells’ function in cancer in literature (6–8), to determine the role of B cell in lung tumorigenesis is of great importance for clinical treatment and prevention post CAR-T cell therapy, which eliminates both transformed and normal B cells in most tissues and organs, including lung, the primary targeting site of intravenous infusion. In the present study, tumor-infiltrating B cells are characterized as a positive predictor for delayed cancer progression, improved therapeutic response, and extended survival in patients with lung cancer, in particular lung adenocarcinoma. In murine lung tumor models, B cell abundance is severely reduced during carcinogenesis, whereas B cell depletion by anti-CD20 antibody significantly promotes the initiation and progression of lung tumor, accompanied with impaired tumor infiltration of immune cells, inhibited phagocytic signaling, and diminished memory B cells and plasma cells. These findings unmask the overall antitumor role of B cells in lung cancer, shedding light on lung cancer pathogenesis and clinical prevention after CAR-T cell therapy.

B cell abundance in tumor was found increased in several papers (21–24), but decreased in others (13–16). In the present study, B cell frequency is severely reduced in both carcinogen-treated lung tissues and lung tumors from various lung cancer models, compared to that in normal lung tissues (**Figure 2**). This discrepancy is probably caused by cancers with different stages used for analysis. The immune cell composition and B cell subpopulations are evolved with tumor progression and therapy, suggesting a switch from tumor-inhibiting to tumor-promoting function (31, 32). For example, a recently identified novel proangiogenic B cell subset, Breg, and Treg are enriched along with cancer progression, while CTL decreased (33). This hypothesis fits well with the conflicting observations of both positive and negative prognostic roles of B cells in human cancers (6–8). In addition, carcinogen-induced oncogenic mutation status also has an impact on B cell infiltration into tumor, as evidenced by lower B cell frequency in lung cancer patients with Kras mutation (34, 35). These data indicate that both infiltration level and overall role of B cells in cancers are dependent on tumor stages, mutations, and therapeutic treatments.

Besides antibody production, B cells indirectly restrict lung cancer initiation and progression through regulation of other immune cells. Although comparable activation status of CD4^+^ and CD8^+^ T cells in lung tissues and lung tumors in both groups, there is a remarkable reduction of tumor infiltration of T cells by B cell depletion (**Figures 4-5B**), resulting in a net inhibition of T cell immunity in tumor microenvironment. In addition, B cells limit Treg function in lung cancer, substantiated by higher Treg frequency by B cell depletion in tumor, lung, TDLN, and spleen (**Figure 5F**; **Supplementary Figure S5**; Ref 36). Other immune cells responsible for ADCC and phagocytosis, including NK, macrophage, dendritic cell, and monocyte, are repressed in lung tumors by anti-CD20 antibody treatment (**Figures 5B, C**). Moreover, the classic and non-classic “don’t eat me” signals are boosted by B cell depletion, whereas “eat me” signals suppressed (**Figure 6**). These data indicate B cells restrain lung tumor initiation and progression probably through activating T cell response, NK-mediated ADCC, and APC-dependent phagocytosis.

Tumor-infiltrating B cell frequency not only predicts better survival in patients with lung cancer, but also therapeutic response, including PD-1/PD-L1 immune checkpoint blockade (37–39), which is in part explained by higher PD-L1 expression level in B cell-enriched tumors (40). Another reason is that B cell can reprogram the tumor microenvironment by recruitment of immune cells, turning “cold” lung tumor to “hot” to improve the efficacy of immunotherapy (**Figure 5**). Furthermore, positive predictable roles of B cells in lung cancer are also found in the context of chemotherapy, primary therapy, and follow-up treatment (**Supplementary Figure S3**). These data demonstrate B cell as a novel prognostic biomarker for better therapeutic response and survival in patients with lung cancer.

In summary, we find tumor-infiltrating B cell abundance is positively associated delayed cancer progression, improved therapeutic response, and extended survival in patients with lung cancer. In murine lung tumor models, B cell frequency is severely reduced, whereas B cell depletion by anti-CD20 antibody significantly accelerates the initiation and progression of lung tumors, which is mediated by repressed tumor infiltration of immune cells, inhibited phagocytic signaling, and diminished memory B cells and plasma cells. Taken together, these findings discover the overall antitumor role of B cells in lung cancer, providing novel insights into cellular mechanisms underlying lung cancer pathogenesis and clinical prevention post CAR-T cell therapy.

## Data availabiltity statement

The datasets presented in this study can be found in online repositories. The names of the repository/repositories and accession number(s) can be found in the article/**Supplementary Material**.

## Ethics statement

The animal study was reviewed and approved by Animal Ethics Committee of Wuhan University of Science and Technology.

## Author contributions

FS, TZ, and WH conceived and designed the study. HW, CC, LG, JL, YY, ML, and YC performed the experiments. HW, CC, XZ, and YL analyzed the data. FS, HZ, and XL wrote the manuscript. All authors contributed to the article and approved the submitted version.

## Funding

This study was supported by grants from the National Natural and Science Foundation of China (82103621, 2017YFE0129100), China Postdoctoral Science Foundation (2021M702538), Department of Education of Hubei Province (B2021023), Department of Science and Technology of Hubei Province (2019ACA168), and Zhongnan Hospital of Wuhan University (LCYF202208, ZNJC202015, PTXM2021019, and cxpy2019088).

## Acknowledgments

The authors thank Baiyin Yuan and Jianhong Sun for their technical assistance and support in animal experiments, Xiang Zhou and Yao Xu for their critical and constructive advice and feedback.

## Conflict of interest

The authors declare that the research was conducted in the absence of any commercial or financial relationships that could be construed as a potential conflict of interest.

## Publisher’s note

All claims expressed in this article are solely those of the authors and do not necessarily represent those of their affiliated organizations, or those of the publisher, the editors and the reviewers. Any product that may be evaluated in this article, or claim that may be made by its manufacturer, is not guaranteed or endorsed by the publisher.
