# Supplementary material for: B cell deficiency promotes the initiation and progression of lung cancer
Source: Front Oncol. 2022 Sep 29;12:1006477. doi: 10.3389/fonc.2022.1006477 (PMC9556970; doi:10.3389/fonc.2022.1006477)
Supplement: Supplementary file 1 [file DataSheet_1.pdf]

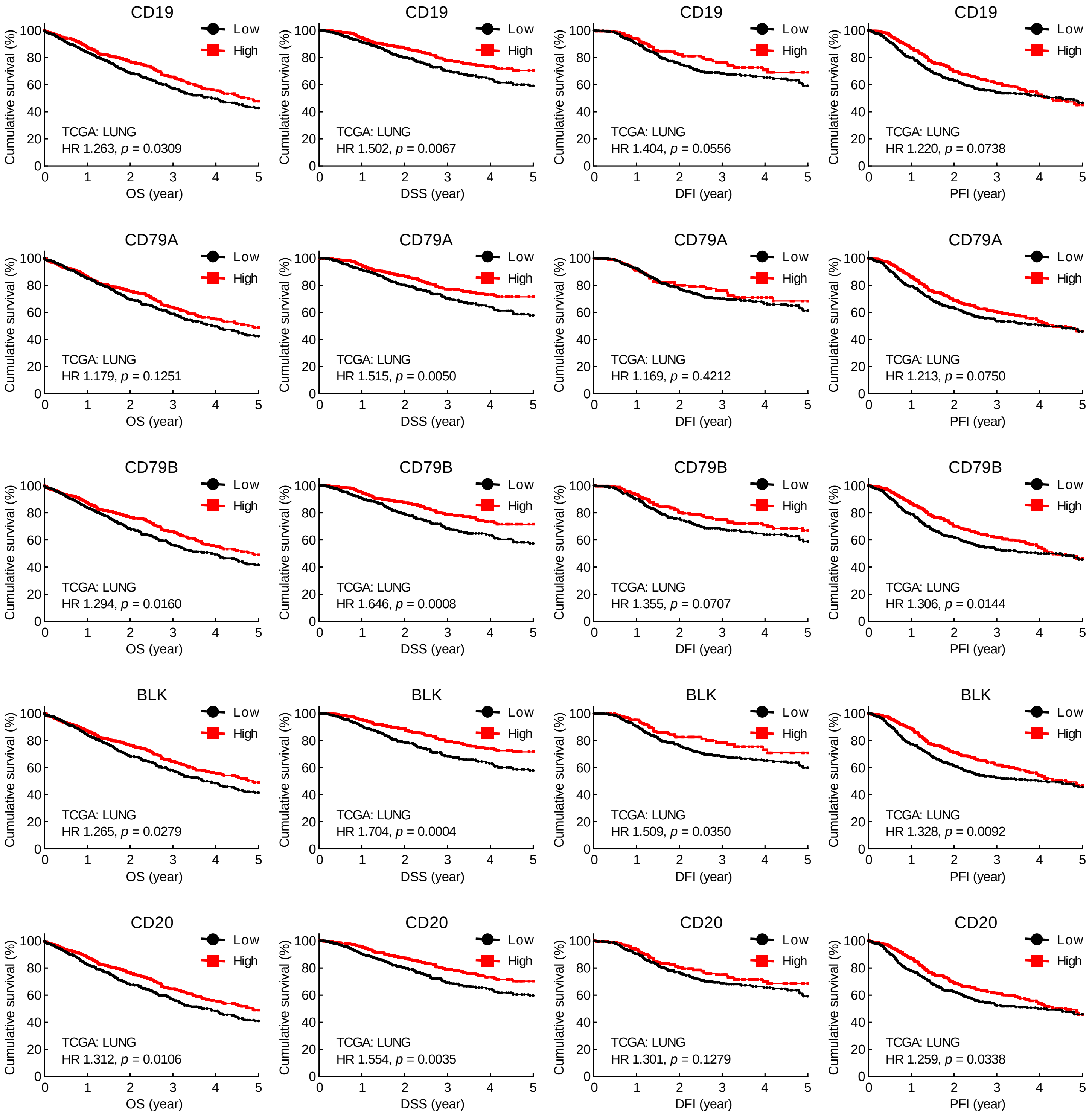

FIGURE S1 Specific B cell markers are positively associated with patient survival in lung cancer. TCGA LUNG data showing positive correlation between specific B cell markers (CD19, CD79A, CD79B, BLK, and CD20) and overall survival (OS), disease specific survival (DSS), disease free interval (DFI), and progression free interval (PFI) in lung cancer. The hazard ratio (HR) and the log-rank *p* value for Kaplan-Meier curve were shown in plots.

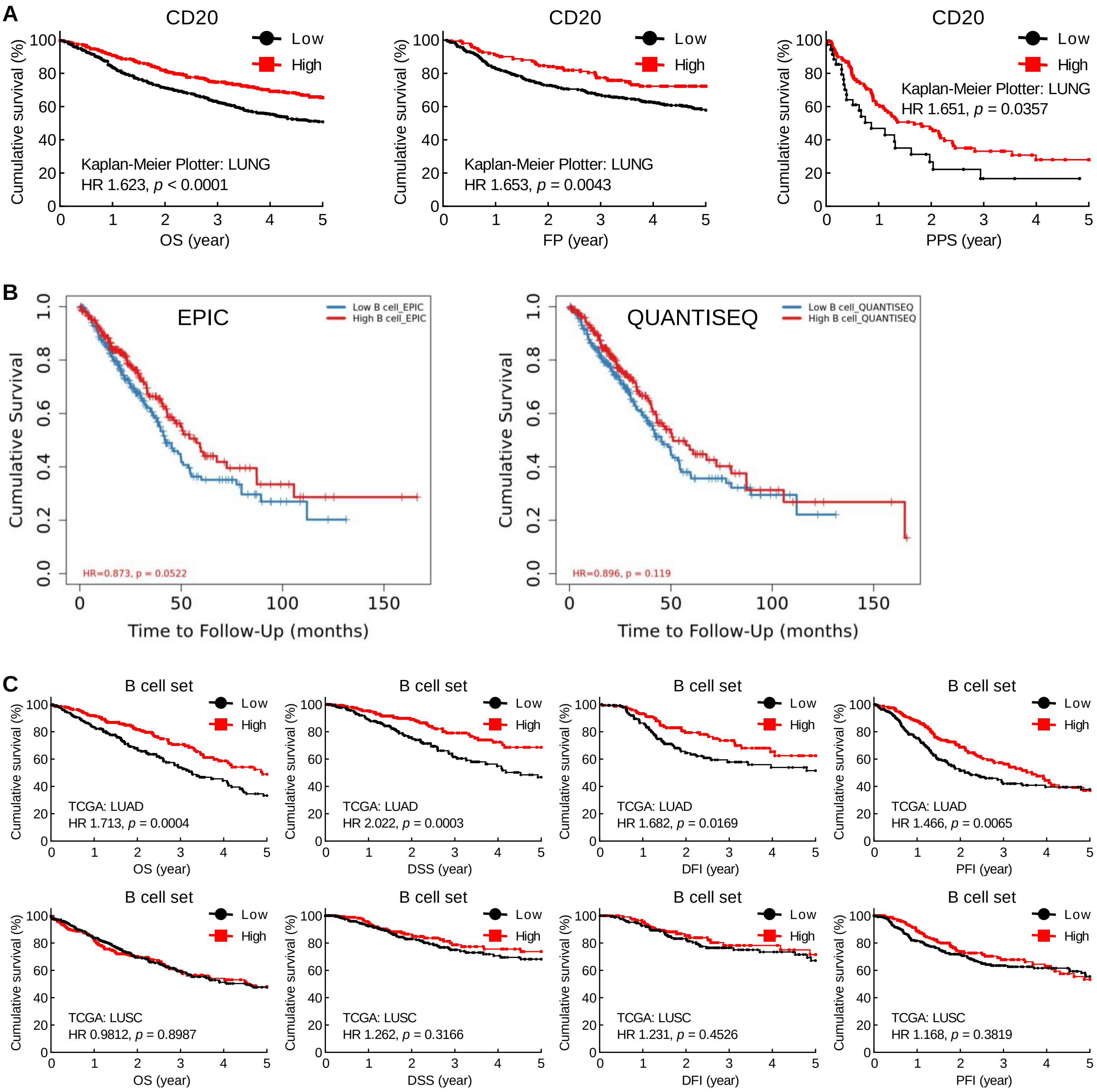

FIGURE S2 B cells are positively associated with patient survival in lung adenocarcinoma. **(A)** Kaplan-Meier Plotter data showing positive association of CD20 expression with overall survival (OS), first progression (FP), and post-progression survival (PPS). **(B)** Kaplan-Meier curves exhibiting a trend of positive association between B cell infiltrates and patient survival in lung adenocarcinoma (LUAD) by EPIC and QUANTISEQ algorithms. The infiltration level is equally divided into low and high levels. **(C)** TCGA LUNG data showing positive correlation between B cells and overall survival (OS), disease specific survival (DSS), disease free interval (DFI), and progression free interval (PFI) in lung adenocarcinoma (LUAD, upper panel) but not lung squamous cell carcinoma (LUSC, lower panel). The hazard ratio (HR) and the log-rank *p* value for Kaplan-Meier curve were shown in plots.

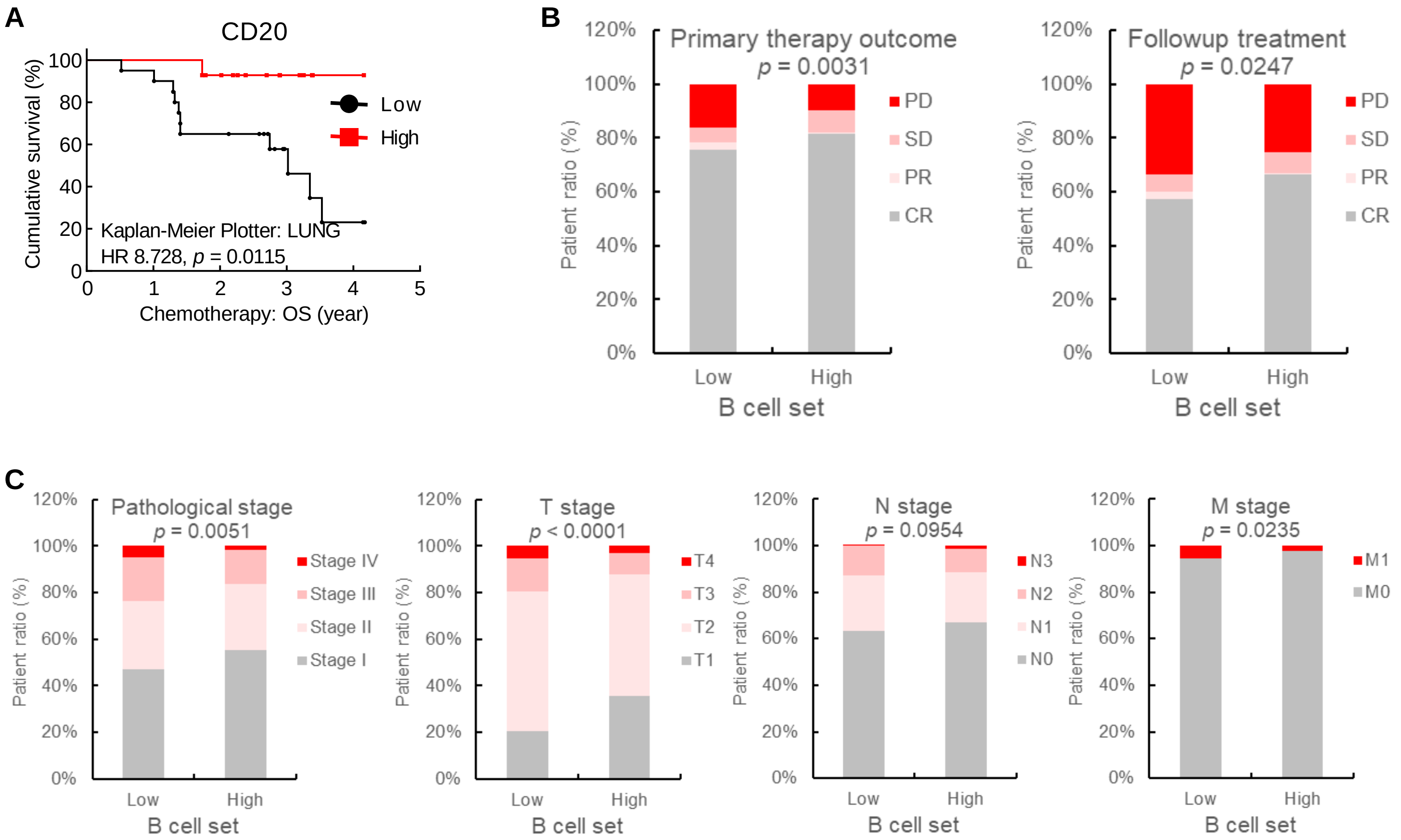

FIGURE S3 High B cell abundance is associated with better therapeutic response and delayed progression in lung cancer. **(A)** Kaplan-Meier Plotter data showing better overall survival (OS) of patient with high CD20 expression level in the context of chemotherapy. **(B)** TCGA LUNG data showing improved clinical outcomes in patient with high tumor infiltration of B cell. **(C)** TCGA LUNG data showing delayed cancer progression in patient with high tumor infiltration of B cell. PD: progressive disease; SD: stable disease; PR: partial remission/response; CR: complete remission/response. The hazard ratio (HR) and the log-rank *p* value for Kaplan-Meier curve were shown in plot **(A)**. Chi-square test was performed in **(B-C)**.

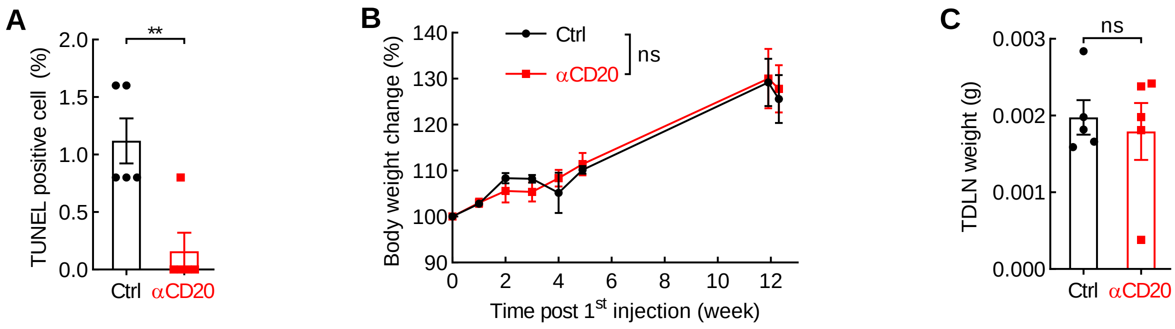

FIGURE S4 B cell depletion has minimal impact on body weight in lung cancer. **(A)** TUNEL assay showing decreased tumor cell apoptosis in mice with αCD20-mediated B cell depletion (n = 5). **(B-C)** No significant effect of αCD20-mediated B cell depletion on body weight (**B**, n = 5) and TDLN weight (**C**, n = 5). TDLN: mediastinum lymph node. Student's *t* test (two tailed, unpaired) was performed. Data represented means ± SEM. ns, not statistically significant.

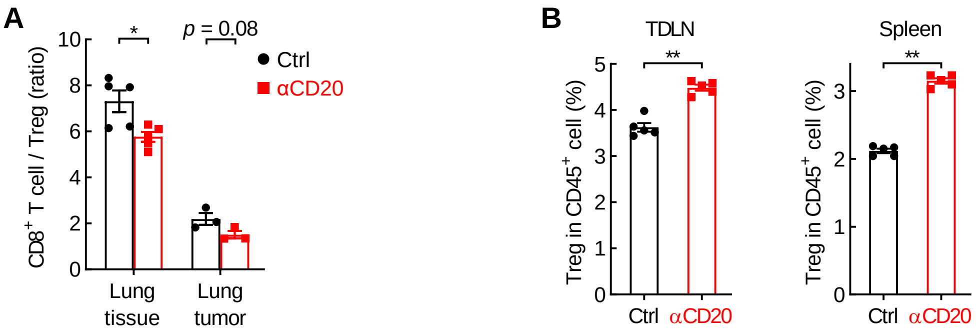

FIGURE S5 Treg cell is elevated by B cell depletion in lung cancer. **(A)** FACS data showing decreased ration of $CD8^+$ T cell versus Treg cell in both lung tissue (n = 5) and lung tumor (n = 3) by αCD20-mediated B cell depletion. **(B)** FACS data showing enriched Treg cell in both TDLN and spleen by αCD20-mediated B cell depletion (n = 5). Student's *t* test (two tailed, unpaired) was performed. Data represented means ± SEM. $*p < 0.05$; $**p < 0.01$.

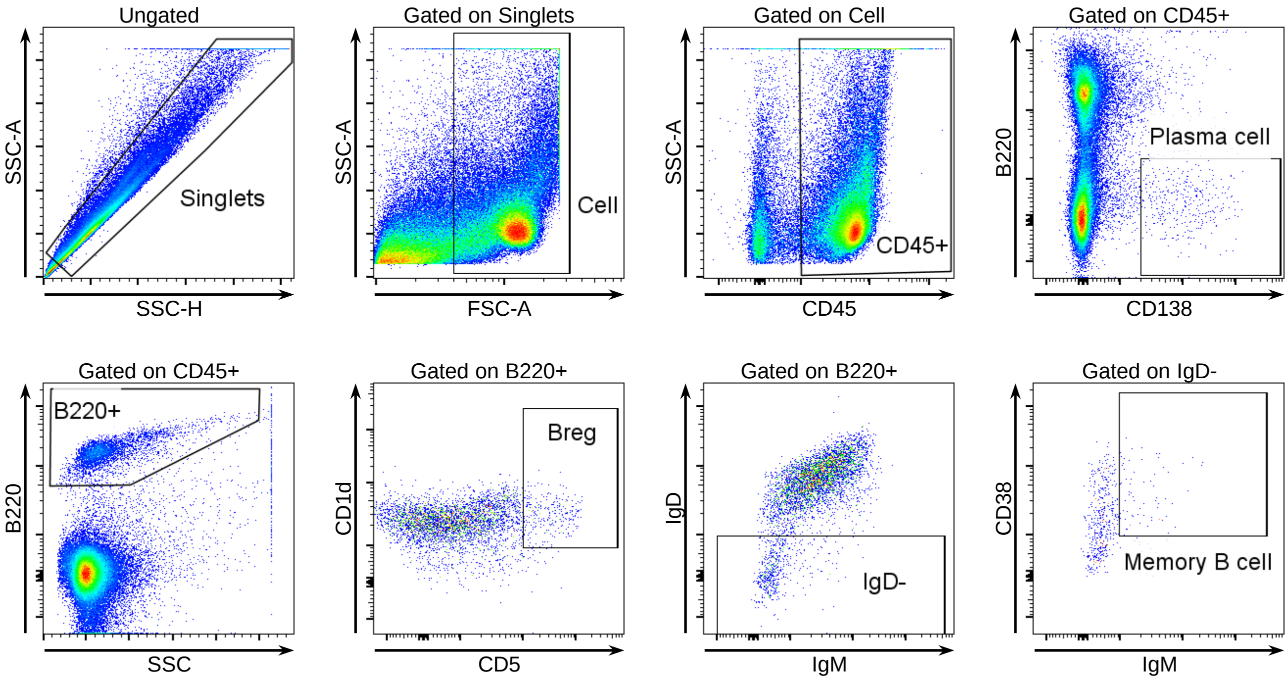

FIGURE S6 FACS gating strategy for B cell subsets in murine tissues. Gating strategy for total B cell (B220+), Breg (B220+CD1d+CD5+), memory B cell (B220+IgD-IgM+CD38+), and plasma cell (B220-CD138+) from CD45+ immune cells.

**TABLE S1** Antibody list					
**Antibody**	**Clone**	**Cat. No.**	**Company**	**Usage**	**Purpose**
Anti-B220 BV510	RA3-6B2, rat IgG2a, κ	103248	Biolegend, San Diego, CA, USA	2.5 µl per sample	FACS
Anti-CD11b PE	M1/70, rat IgG2b, κ	12-0112-82	Thermo Fisher Scientific, Waltham, MA, USA	0.625 µl per sample	FACS
Anti-CD11c APC-eFluor780	N418, armenian hamster IgG	47-0114-82	Thermo Fisher Scientific, Waltham, MA, USA	2.5 µl per sample	FACS
Anti-CD138 PE	281-2, rat IgG2a, κ	142504	Biolegend, San Diego, CA, USA	1.25 µl per sample	FACS
Anti-CD16/CD32	93, rat IgG2a, λ	14-0161-85	Thermo Fisher Scientific, Waltham, MA, USA	1.0 µl per sample	FACS
Anti-CD1d PerCP-Cy5.5	1B1, rat IgG2b, κ	123514	Biolegend, San Diego, CA, USA	1.25 µl per sample	FACS
Anti-CD24 BV510	M1/69, rat IgG2b, κ	101831	Biolegend, San Diego, CA, USA	2.5 µl per sample	FACS
Anti-CD25 PE	PC61.5, rat IgG1, λ	12-0251-81	Thermo Fisher Scientific, Waltham, MA, USA	0.625 µl per sample	FACS
Anti-CD38 APC	90, rat IgG2a, κ	102712	Biolegend, San Diego, CA, USA	1.25 µl per sample	FACS
Anti-CD3e APC-eFluor780	145-2C11, armenian hamster IgG	47-0031-82	Thermo Fisher Scientific, Waltham, MA, USA	5 µl per sample	FACS
Anti-CD3e PE	145-2C11, armenian hamster IgG	12-0031-83	Thermo Fisher Scientific, Waltham, MA, USA	2.5 µl per sample	FACS
Anti-CD4 APC-eFluor780	RM4-5, rat IgG2a, κ	47-0042-82	Thermo Fisher Scientific, Waltham, MA, USA	0.625 µl per sample	FACS
Anti-CD4 PE-Cy7	RM4-5, rat IgG2a, κ	25-0042-81	Thermo Fisher Scientific, Waltham, MA, USA	1.25 µl per sample	FACS
Anti-CD45 Alexa Fluor 700	30-F11, rat IgG2b, κ	103128	Biolegend, San Diego, CA, USA	0.5 µl per sample	FACS
Anti-CD47 FITC	miap301, rat IgG2a, κ	11-0471-82	Thermo Fisher Scientific, Waltham, MA, USA	2 µl per sample	FACS
Anti-CD5 PE-Cy7	53-7.3, rat IgG2a, κ	100621	Biolegend, San Diego, CA, USA	1.25 µl per sample	FACS
Anti-CD64 PE-Cy7	X54-5/7.1, mouse IgG1, κ	139314	Biolegend, San Diego, CA, USA	2.5 µl per sample	FACS
Anti-CD8a APC	53-6.7, rat IgG2a, κ	17-0081-83	Thermo Fisher Scientific, Waltham, MA, USA	0.625 µl per sample	FACS
Anti-EpCAM BV421	G8.8, rat IgG2a, κ	118225	Biolegend, San Diego, CA, USA	1.25 µl per sample	FACS
Anti-Foxp3 FITC	FJK-16s, rat IgG2a, κ	11-5773-82	Thermo Fisher Scientific, Waltham, MA, USA	2.0 µl per sample	FACS
Anti-Granzyme B FITC	NGZB, rat IgG2a, κ	11-8898-80	Thermo Fisher Scientific, Waltham, MA, USA	0.25 µl per sample	FACS
Anti-IFNγ FITC	XMG1.2, rat IgG1, κ	11-7311-81	Thermo Fisher Scientific, Waltham, MA, USA	1.0 µl per sample	FACS
Anti-IgD APC-Cy7	11-26c.2a, rat IgG2a, κ	405715	Biolegend, San Diego, CA, USA	1.25 µl per sample	FACS
Anti-IgM FITC	RMM-1, rat IgG2a, κ	406506	Biolegend, San Diego, CA, USA	2 µl per sample	FACS
Anti-Ly6C BV650	HK1.4, rat IgG2c, κ	128049	Biolegend, San Diego, CA, USA	1.25 µl per sample	FACS
Anti-Ly6G BV510	1A8, rat IgG2a, κ	127633	Biolegend, San Diego, CA, USA	2.5 µl per sample	FACS
Anti-MerTK Super Bright 436	DS5MMER, rat IgG2a, κ	62-5751-82	Thermo Fisher Scientific, Waltham, MA, USA	5.0 µl per sample	FACS
Anti-MHC-II APC	M5/114.15.2, rat IgG2b, κ	17-5321-82	Thermo Fisher Scientific, Waltham, MA, USA	0.15 µl per sample	FACS
Anti-NKp46 BV421	29A1.4, rat IgG2a, κ	137612	Biolegend, San Diego, CA, USA	2.5 µl per sample	FACS
Anti-PD-L1 Super Bright 780	MIH5, rat IgG2a, λ	78-5982-82	Thermo Fisher Scientific, Waltham, MA, USA	0.3 µl per sample	FACS
Anti-CD20	SA271G2, rat IgG2b, κ	152116	Biolegend, San Diego, CA, USA	200 µg/mouse/time	*in vivo* blockade
Anti-Cleaved Caspase 3	Asp175, rabbit polyclonal	9661	Cell Signaling Technology, Danvers, MA, USA	1:200	IHC
Goat anti-rabbit IgG biotinylated		E0432	Dako, Carpinteria, CA, USA	1:300	IHC
